# Measuring Heat Production from Burning Al/Zr and Al/Mg/Zr Composite Particles in a Custom Micro-Bomb Calorimeter

**DOI:** 10.3390/ma13122745

**Published:** 2020-06-17

**Authors:** Elliot R. Wainwright, Madeline A. Mueller, Kyle R. Overdeep, Shashank Vummidi Lakshman, Timothy P. Weihs

**Affiliations:** Department of Materials Science & Engineering, Johns Hopkins University, Baltimore, MD 21218, USA; mmuell15@jhu.edu (M.A.M.); kyleoverdeep@jhu.edu (K.R.O.); shashankvummidi@jhu.edu (S.V.L.)

**Keywords:** bomb calorimetry, physical vapor deposition, ball milling, particle size, sintering, metal combustion, combustion efficiency

## Abstract

Al:Zr, Al-8Mg:Zr, and Al-38Mg:Zr nanocomposite particles fabricated by physical vapor deposition (PVD) and ball milling were reacted in 1 atm of pure O_2_ within a custom, highly-sensitive micro-bomb calorimeter. The heats of combustion were compared to examine the effect of particle size and composition on combustion efficiency under room temperature and in a fixed volume. All particles yielded ~60–70% of their theoretical maximum heat of combustion and exhibited an increase in heat over composite thin films of similar compositions, which is attributed to an increase in the surface area to volume ratio. The effect of particle size and geometry are mitigated owing to the sintering of the particles within the crucible, implying the importance of particle dispersion for enhanced performance. Vaporization of the metal species may transition between two diffusion flame species (Mg to Al). As Mg content is increased, more vaporization may occur at lower temperatures, leading to an additional stage of sintering. Physically intermixed Al and Mg oxides have been observed coating the surface of the particles, which implies a continuous transition of these vaporization processes. Such nano-oxides imply high vapor-flame combustion temperatures (>2700 K) and suggest viability for agent defeat applications.

## 1. Introduction

Metal fuels, including their more complex composite derivatives, have been studied extensively owing to their potential applications in mining, excavation, propulsion, explosives, and bio- and chemical-agent defeat [1,2,3,4]. For example, spores and chemical agents within a fireball can be destroyed thermally (via heat of combustion) and chemically using neutralizing gases and reactions with oxide products [5,6,7,8,9,10,11]. For these applications, fuels such as Al, Mg, and Zr are commonly utilized given their high volumetric and gravimetric combustion enthalpy [1]. While the process of heterogeneous combustion of pure metal fuels such as Al, Mg, and Zr are well known [12,13,14,15], these processes can be complex and vary with composition, size, environmental gases, and ignition and dispersion methods [1].

Recently, there have been efforts to engineer metal fuels with independently tunable ignition and combustion properties by generating composite particles via high energy ball milling. For example, the average reactant spacing of the Al and Zr species within the microstructure can be varied to both lower their ignition temperature relative to pure Al of similar sizes and tune their ignition temperature [16]. The chemistry and size of the particles can be altered to control the energy and duration of combustion through the formation of oxides and nitrides [17,18,19,20,21,22]. Intermetallic reactions have been utilized in materials of varying compositions and structures [23,24] such as mechanically fabricated foils and particles [25,26,27,28,29] and vapor deposited laminate foils [30,31,32,33,34,35,36,37]. These intermetallic formation reactions consist of exothermic intermixing of the two metallic species and precipitation of ordered intermetallic phases; these processes lead to composite foil/particle ignition and are a subset of a general class of self-propagating high-temperature synthesis (SHS) reactions [24,38,39]. The exothermic formation reactions in these composite metal particulate systems are thought to raise the particles to high temperatures where rapid oxidation/nitridation/combustion begin.

Physical vapor deposition (PVD) offers a means for producing composite Al/Zr particles with uniform composition and size. While expensive to fabricate, PVD particles can serve as model materials for combustion studies. In contrast, mechanical processing of composite powders through ball-milling [40] offers a relatively inexpensive way to fabricate large quantities of reactive composite particles. Thus, reactive/energetic formulations will tend to utilize mechanically processed composite metal fuels in powder or particle form to enhance scalability and effective mixing with oxidizers and binders [41,42,43]. However, ball milled particles may show distributions in particle chemistry, size, and average reactant spacing [22]. Thus, it is important to draw comparisons between materials synthesized under these two methods in order to demonstrate their similarity (or differences) for scale up in a given application.

Previous research on small quantities of ball milled Al:Zr particles has focused on studying their dual-phase combustion, which proceeds with Al vapor phase oxidation and Zr condensed state nitridation followed by Zr oxidation [16,44,45]. However, these studies lacked a measure of combustion efficiency, which is performed for the first time at small scales in this study. Other work has added Mg to generate an Al/Mg/Zr composite in order to assess the effect on combustion efficiency, maximum temperatures, burn rate, and so on [21,46]. Mg has also been added to Al and has been shown to decrease ignition delays and increase burn rates [47,48,49,50,51]. Mg may also provide oxides desirable for agent defeat [52,53]. Thus, understanding the effects of adding Mg to Al/Zr composites and quantifying the effects of increasing Mg content on combustion efficiency are desired.

In this study, the heats of combustion of Al/Zr and Al/Mg/Zr composite particles are characterized via bomb calorimetry. Novel PVD particles and ball mill particles with several different Al–Mg–Zr compositions are synthesized and reacted within a custom-made, highly sensitive micro-bomb calorimeter in order to assess the varying effects of particle composition, size, and geometry on heat output and product formation during combustion. The particles are ignited in 1 atm of O_2_ to compare the combustion efficiency of the various composites as a function of composition without over-driving the combustion, as is done under typical bomb calorimetry conditions (i.e., 30 atm of O_2_). Comparisons with previously published data of the combustion efficiency of reactive thin film systems of the same composition are made. This work provides new comparisons with larger scale explosive testing of powders [21], which effectively provides dynamic bomb calorimetry measurements in a much larger fixed-volume chamber.

## 2. Materials and Methods

### 2.1. Particle Synthesis

**Figure 1:**
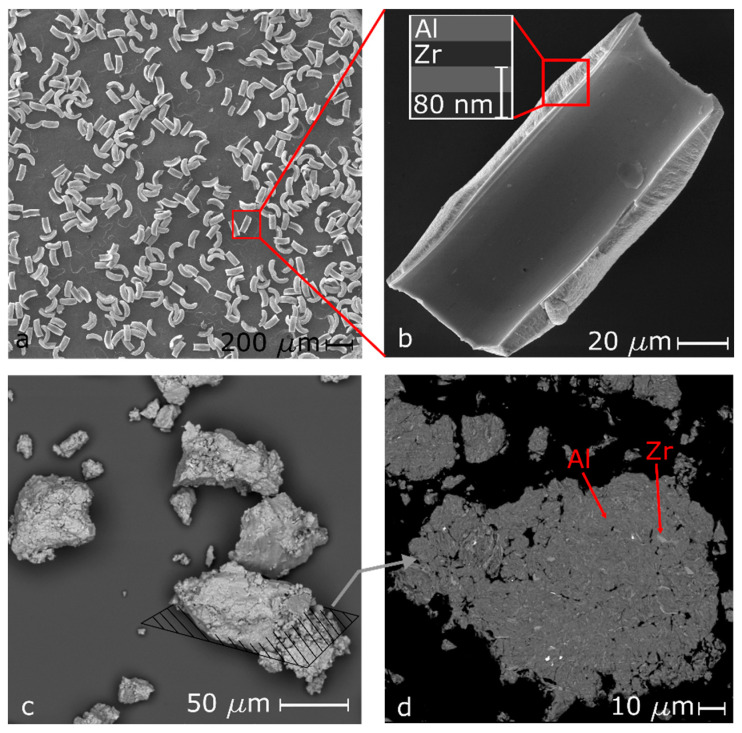
(**a**) Crescent-shaped Al:Zr physical vapor deposition (PVD) particles with lengths approximately equal to three times their widths. The particles are uniform in geometry for a given mesh size. (**b**) A single Al:Zr PVD particle. A schematic of the bilayer structure of each particle is shown within the inset. (**c**) Several ball milled Al:Zr particles exhibiting a rough and granular morphology with lines representing a cross-sectional slice, an example of which is shown in part (**d**). (**d**) A representative cross-section of an Al:Zr particle taken via backscatter scanning electron microscope (SEM), exhibiting Zr inclusions within an Al matrix.

To make the PVD particles, a repeating Al/Zr or Al–Mg/Zr bilayer structure is magnetron sputtered onto nylon mesh substrates using a rotating, water-cooled carousel. A similar sputtering process of nanolaminate foils is described in [19] and was modified as described below for this study to obtain the compositions of interest. PVD particles were fabricated using 99.7 at.% Zr targets with either Al–1100 (minimum 99 at.% Al), Al–8at.%Mg, or Al–38at.%Mg targets (Plasmaterials Inc., Livermore, CA, USA) [46]. The powers to the two cathodes, located on either side of the chamber, were adjusted to achieve a 50/50 atomic ratio between Zr and the desired Al or Al–Mg chemistry (denoted Al:Zr, Al–8Mg:Zr, and Al–38Mg:Zr). The rotation rate was varied to obtain a bilayer thickness of 80 nm, and the total number of rotations or bilayers was controlled to obtain a maximum thickness of 60 µm for each composition. Particle size variation was studied by sputtering Al:Zr particles with maximum thicknesses of 20, 40, and 60 µm. After deposition, the particles were gently removed from the mesh substrates underwater via mechanical agitation and then dried. The shape of the mesh resulted in particles with a half-pipe shaped morphology, as shown in Figure 1a, and the cylindrical nature of the nylon wires and the angles of deposition resulted in particles that varied slightly in total thickness as one moves across the width of each particle, as seen in Figure 1. The width of each particle was equal to the diameter of the nylon fiber and the length was ~3× the diameter, dictated by the square pattern of the mesh substrate (Figure 1b).

**Figure 2:**
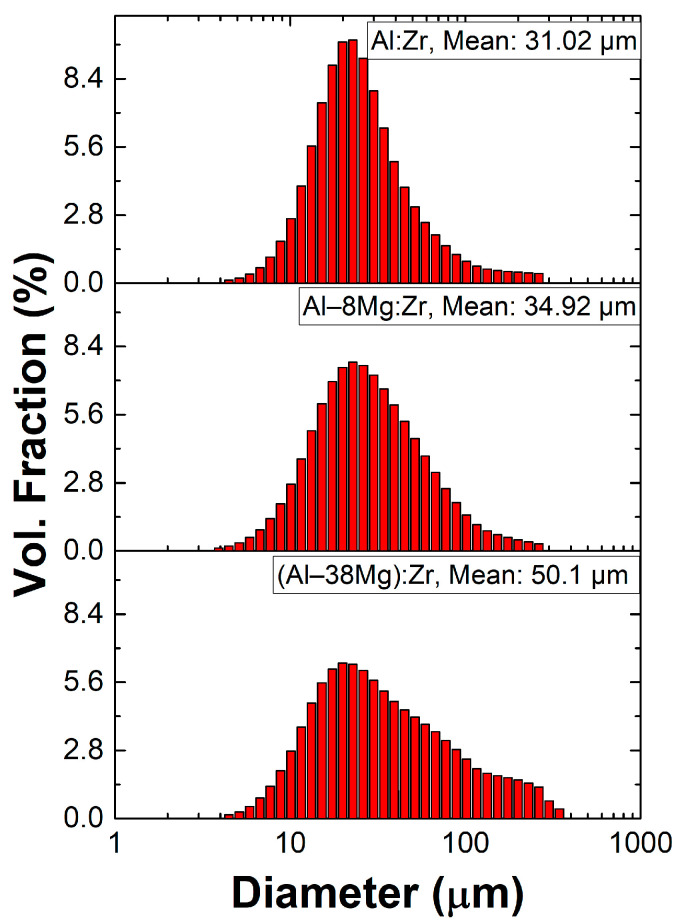
Ball milled particle size distributions for Al:Zr, Al-8Mg:Zr, and Al-38Mg:Zr compositions.

Separate particles of similar compositions were also fabricated by milling powders of Zr (Atlantic Equipment Engineers, Upper Saddle River, NJ, USA, −50 mesh) and combinations of Al (Alfa Aesar, Ward Hill, MA, USA, −325 mesh), Al–8Mg (Goodfellow, Huntingdon, UK, −230 mesh), and Al-50Mg (Skylighter, Purcellville, VA, USA, −325 mesh) in a 8000D Shaker Mill (SPEX, Metuchen, NJ, USA) for 1 h with a ball to powder ratio (BPR) of 10 and with hexane as a process control agent. To obtain the Al–38Mg:Zr chemistry, particles were milled in a single step using the appropriate combination of both Al and Al–50Mg powders. The resulting milled particles were granular in nature with rough, jagged edges, as shown in Figure 1c. A representative cross-sectional view of the interior of a ball milled particle is shown in Figure 1d, where the dark matrix is Al, the lighter inclusions are Zr, and the black background is epoxy. The particle size distributions of the resulting ball milled particles of different chemistries are shown in Figure 2, measured using laser diffraction (Horiba LA-950, Edison, NJ, USA isopropanol suspension). Both types of particles were compared to 40 µm thick, ~11 mm wide, ~50 mm long sputter deposited foils of the same composition, using data from [46].

### 2.2. Custom Particle Bomb Micro-Calorimeter Design and Measurement

**Figure 3:**
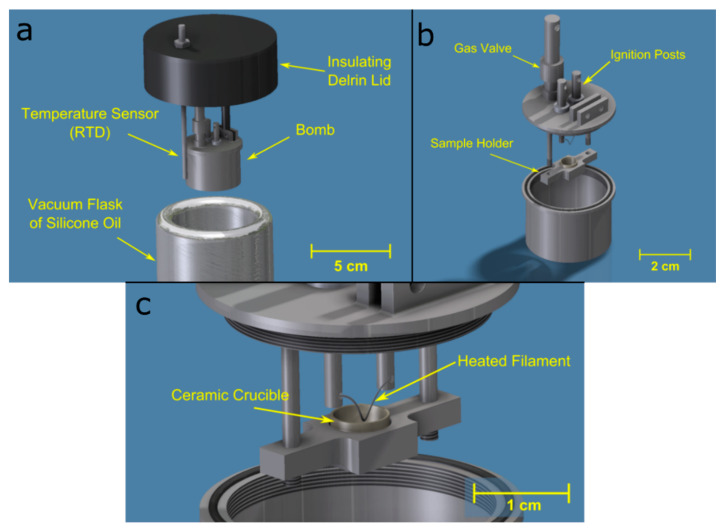
(**a**) Major components of the Ti particle bomb calorimeter assembly, including a vacuum flask of silicone oil, an resistance temperature detector (RTD), and a Delrin lid for insulation. (**b**) The environment of the bomb is controlled via the gas valve welded to the lid, and ignition is achieved by connecting leads from the power supply to the ignition posts. (**c**) Particle samples are contained in a ceramic crucible that is raised until the sample is in contact with a filament that is resistively heated for ignition.

The design of the custom particle calorimeter follows that of the custom thin-film micro-calorimeter described in [54], and has been utilized in earlier work with Al-based thermite reactions [55]. Figure 3a depicts the particle calorimeter assembly. High sensitivity is achieved with this calorimeter by constructing the bomb out of Ti and by minimizing the mass and volume of the bomb itself, as well as the liquid bath. This bomb has half the volumetric capacity of the referenced thin-film micro-calorimeter, allowing for a higher calorimetric sensitivity of 135 J·K^−1^. The design of the bomb itself, shown in Figure 3b, has a gas valve, two posts that serve as terminals for electrical connections for ignition, and a ceramic particle sample holder, as shown in Figure 3c. A V-shaped 0.127 mm Nichrome filament (alloy composition of Chromel C 60% Ni, 16% Cr, and 24% Fe, from McMaster-Carr, Los Angeles, CA, USA) placed between the electrical terminal posts is used to ignite the powders in the holder. A bath of silicone oil was used to increase the sensitivity of the measurements owing to the lower heat capacity of silicone oil (1.59 J·g^−1^·K^−1^) compared with that of water (4.18 J·g^−1^·K^−1^) [56]. After temperature equilibration, a power supply is set to 10 V and then triggered to resistively heat the filament and ignite the particles. The energy added to the system from the resistively heated wire is measured and subtracted from the total heat that is calculated from the time rise within the silicone oil bath.

Table 1 lists the sample masses for all three compositions that are required to consume 100% of the available O_2_ in the calorimeter assuming complete combustion in 1 atm of 99.999% pure O_2_. To ensure that excess oxygen was available during combustion, only 17 mg of each sample (PVD or ball milled particles) was reacted in the calorimeter in O_2_. In typical bomb calorimetry experiments, 30 atm of O_2_ is used, which drives particles to artificially high levels of combustion efficiency relative to that achievable in applications. Thus, 1 atm was utilized in order to assess relative material performance under ambient conditions, but pure O_2_ was utilized rather than air to allow for more sample mass for a given degree of combustion. The testing of larger sample masses is thought to enhance the signal to noise ratio. The temperature rise data of the bomb were measured and the heat output was calculated using LabVIEW; the methodology described in [54,57]. Each sample was tested three times to obtain average values for heat outputs.

### 2.3. Particle Characterization

Pre- and post-reaction samples were analyzed with a Tescan Mira GMU III scanning electron microscope (SEM) under backscatter conditions. In addition, energy-dispersive X-ray spectroscopy (EDS) was performed using an EDAX TEAM Analysis System to determine the post-reaction presence of oxides. Post-reaction particles were also analyzed via powder X-ray diffraction at 45 kV and 40 mA with Cu-Kα radiation (Philips X’pert MRD diffractometer, Malvern, UK). The ignition and dispersion of the burning particles were observed using the calorimeter crucible housing assembly (Figure 3c) in a larger, 22.4 L vacuum chamber, pressurized to 1 atm of O_2_, and an NAC Memrecam HX-6 High-Speed Camera (Tokyo, Japan) recording at 3000 frames per second. The high-speed videography allowed for the observation of the extent of particle dispersion under conditions similar to those of the much smaller and sealed bomb calorimeter.

## 3. Results

**Figure 4:**
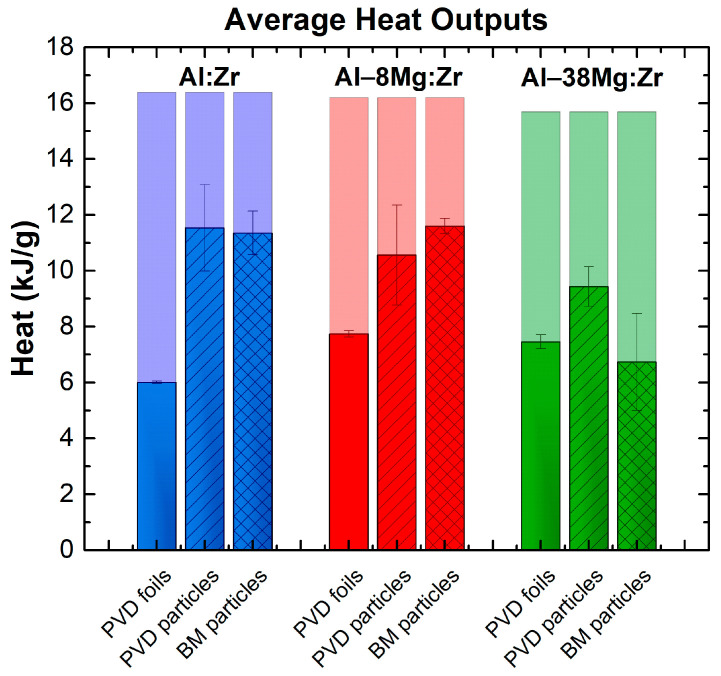
Heat output in 1 atm of O_2_ for 40 µm thick PVD and ball milled (BM) particles, compared with data for 40 µm thick PVD foils, also in 1 atm of O_2_, for each of the three chemistries of interest. Theoretical heat maxima are denoted by lighter background bars. Heat data for PVD foils were originally presented in [46].

The heats of combustion of 40 µm thick PVD particles and ball milled particles in 1 atm of O_2_ are presented in Figure 4 for the three compositions. The standard deviation bars are generated by running each bomb calorimetry test in triplicate. The theoretical values (light background bars) were calculated by summing standard enthalpies of formation for the thermodynamically preferred Al, Mg, and Zr oxides that can form given the atomic fraction of each element. Data for 40 µm thick PVD foils of the same compositions, also burned in 1 atm of O_2_, are included from [46] as well. The Al:Zr and Al-8Mg:Zr particles, whether ball milled or sputter deposited, all released ≈11.3 kJ·g^−1^ of heat and typically fell within one standard deviation of each other. This value represents a 46% improvement over the 40 µm thick PVD foil, particularly in the Al–8Mg:Zr case. For the Al–38Mg:Zr composition, the PVD particles produced only 26% more heat than the PVD foils, and the ball milled particles produced 20% less heat than the foils on average. This is the only case in which the ball milled materials do not perform within one standard deviation of the PVD particles.

The heat produced by Al:Zr PVD particles of various sizes is presented in Table 2 and shows that, in this experimental configuration, particle size has a negligible effect on heat output, despite changes in the surface-area-to-volume ratios (SA/V). The SA/V values were approximated by treating the PVD particles as half-pipes of various thicknesses, using the expression:

(1) $$\frac{SA}{V}=\frac{2tl+\left(\pi r_{2}^{2}-\pi r_{1}^{2}\right)+l\pi r_{1}+l\pi r_{2}}{\left(\pi r_{2}^{2}-\pi r_{1}^{2}\right)l}$$

where t is the thickness of the particles; l is the length of the particles; and $r_{1}$, $r_{2}$ are the inner and outer radii, respectively. Combustion efficiency should be enhanced as the SA/V increases, implying that the particle size effect is being mitigated in these experiments in some way.

**Figure 5:**
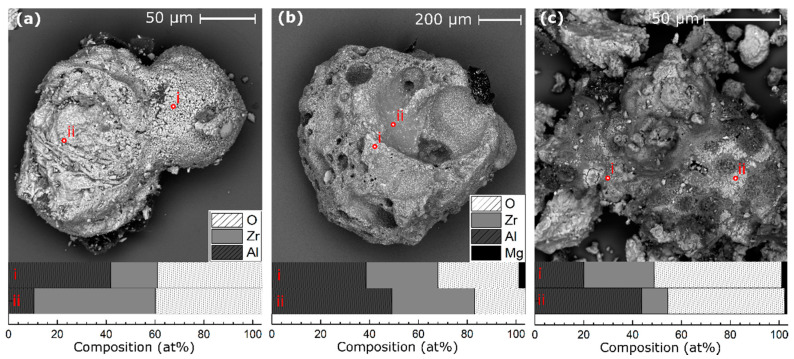
Representative backscatter SEM images of large sintered particles collected post-reaction from the bomb calorimeter crucible. (**a**) Al:Zr PVD 40 µm sintered particles; (**b**) Al–8Mg:Zr PVD 40 µm sintered particles; (**c**) Al–8Mg:Zr ball milled sintered particle(s).

**Figure 6:**
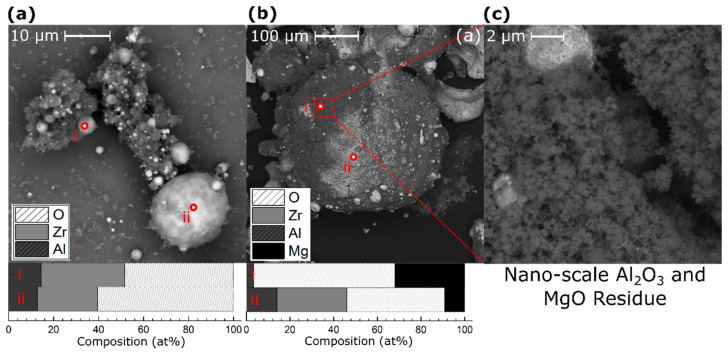
(**a**) Backscatter SEM images of several PVD Al–8Mg:Zr spherical particles collected from from the bottom of the calorimeter chamber, outside of the reaction crucible. (**b**) Backscatter SEM image of a sintered Al–38Mg:Zr PVD particle found inside the crucible and the corresponding EDS scans, showing the presence of Mg. (**c**) A zoomed-in view of the intermixed nano-scale Al_2_O_3_ and MgO residue coated on the surface of the particles found within the crucible.

SEM was performed on post-reacted samples collected from both within the reaction crucible and outside of the crucible in the bottom of the closed bomb chamber. Significant particle sintering was observed for both PVD and ball milled particles and for all three compositions. Figure 5 displays representative cases of Al–8Mg:Zr PVD and ball milled particles collected from within the reaction crucible. These sintered combustion products are much larger than the original particles and have rounded surfaces with varying degrees of roughness, implying they partially melted during the intermetallic formation and combustion reactions. The particles in Figure 5 demonstrate varying levels of oxidation and depletion of Mg relative to the starting value, as measured by EDS point scans. Particles collected from the bottom of the calorimeter chamber show high levels of oxidation, such as the products of 40 µm thick Al–8Mg:Zr PVD particles shown in Figure 6a. The particles in Figure 6 also exhibit high levels of oxidation on their surfaces (50–60 at.% O_2_, as measured by EDS point scans) and have a spherical morphology implying they became molten during reaction. Mg was not detected in significant fractions within these particles. These spherical reaction products are common for all particle types, compositions, and sizes, and are more commonly found outside of the reaction crucible. In addition, the spherical particles are coated by fine alumina residue, suggesting flame temperatures above the boiling point of Al (2743 K). A representative Al–38Mg:Zr PVD particle, shown in Figure 6b, collected from within the crucible, exhibits similar residue, which also contains Mg. A higher magnification image of this soot (shown in Figure 6c) has a fluffy, fractal-like structure on the nanometer-scale. Other examples of spherical particles are shown in the Supplementary Materials, Figures S1 and S2. Products like those shown in Figure 5 and Figure 6 were observed for all particle sizes and compositions.

**Figure 7:**
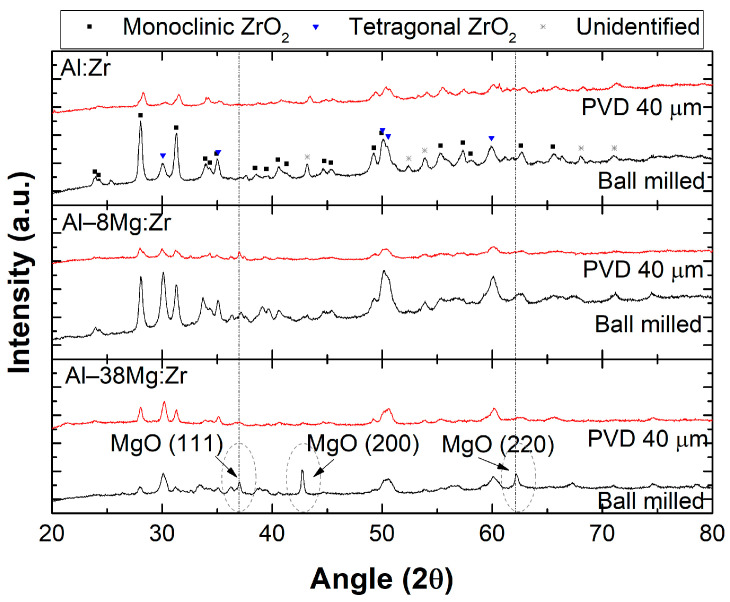
X-ray diffraction data for Al:Zr, Al–8Mg:Zr, and Al–38Mg:Zr particles collected within the reaction crucible.

X-ray diffraction was performed on particles collected from within the reaction crucible for both particle types and for all three compositions. There was an insufficient volume of collected products from outside the crucible in the bottom of the bomb chamber to perform X-ray diffraction (XRD) analysis. The diffraction patterns are shown in Figure 7. The data are presented in Figure 7 without background subtraction. A low signal to noise ratio is common in the PVD cases owing to the small number of particles that could be collected for analysis. Several peaks in Figure 7 that could not be identified are noted. Of particular note is the abundance of ZrO_2_ in monoclinic and tetragonal phases, and the increasing presence of MgO in the Al–8Mg:Zr and Al–38Mg:Zr samples, respectively. Given ZrO_2_ transitions from a monoclinic phase to a tetragonal phase between 1500 and 1200 K upon cooling, the presence of the high-temperature tetragonal phase suggests some particles cool rapidly following combustion at elevated temperatures [58].

**Figure 8:**
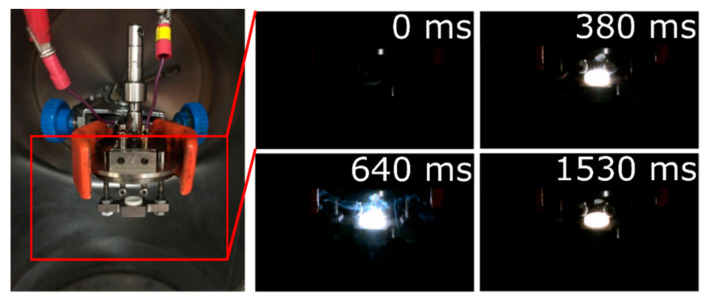
Still frames from a high-speed video of 40 µm Al:Zr PVD particles reacting in a larger chamber with 1 atm of O_2_ and held in the assembly used during the bomb calorimeter experiments (Figure 3c).

Still frames from a high-speed video of 40 µm Al:Zr PVD particles reacting in a chamber purged and filled with 1 atm of O_2_ shows that a small vapor cloud forms above the crucible during the reaction (Figure 8). The vapor appears to be localized in or near the crucible, implying that dispersion of the particles may be limited. The presence of Al, AlO, and MgO has been detected concurrently via spectroscopy in other work with Al/Zr and Al/Mg/Zr reactive composites burning in air (see Supplementary Materials, Figure S3); however, when it was attempted on the particles in the configuration described in Figure 8, the signal was poor owing to the aforementioned lack of particle dispersion from the crucible. The vapor in Figure 8 likely consists of Al and AlO, as has been observed in other experimental conditions [45].

## 4. Discussion

### 4.1. Particle Geometry Effects

Under normal bomb calorimetry conditions (30 atm of O_2_), the rate and general processes of the reactions do not have a strong influence over the combustion efficiency, as reactions generally proceed to completion. However, given that these experiments are completed in only 1 atm of O_2_, the size of particles will partially determine their ability to react to completion before quenching, and so on. The results in Figure 4 suggest that particles combust more effectively than foils with the same composition. This increase in efficiency is demonstrated for all three PVD particle compositions and for two of the ball milled compositions (Al:Zr and Al–8Mg:Zr), and is the result of the significant increase in the SA/V, as expected. For particles of varying size, the combustion efficiency typically improves as particle size decreases, as long as the particles are not so small that surface passivation reduces the active metallic content and, therefore, the particle’s energy density [59,60].

The heats of combustion data presented in Figure 4 and Table 2 imply that, under these experimental conditions, size has a small effect on combustion efficiency for the particles and that they are achieving 60–70% of their theoretical maximum. These values could be even higher, but are mitigated by the specific combination of these fuels and two experimental factors. First, the limited dispersion of particles from the ceramic crucible, shown in Figure 8, causes them to sinter (Figure 5) and impedes oxygen transport. Sintering commonly decreases the effective surface area of burning metals [61,62,63], which would explain both the lack of an initial particle size effect on heat output and the lower heat output compared with theory. Second, when reacting particles are dispersed from the crucible, the relatively small volume of the calorimeter chamber may limit the time for particles to combust before they impact the relatively cool walls of the calorimeter, which can prematurely quench the reaction. These quenching effects may be indirectly indicated by the 2–3× higher concentration of Al remaining in the post-reaction particles within the calorimeter (10–20 at.%) compared with similar particles that were burned outside the chamber in air (~5 at.%) [45], and the presence of the high temperature ZrO_2_ monoclinic phase. The former implies that the particles within the calorimeter did not spend enough time at high temperatures to sufficiently boil Al out of the molten composite powders and the latter implies that they were rapidly quenched from high temperature. Despite these limitations, it should be noted that typical bomb calorimetry is completed under 30 atm, and when tests on a pure Al fuel of smaller sizes (~3 µm mean size) were performed at 1 atm in our custom bomb, there was no ignition or combustion of the Al powders, implying that the utilization of intermetallic formation reactions for ignition benefits their reaction performance in this test environment. For completeness, it is noted that all of the particles tested here ignite at similarly low temperatures ranging from 600 to 700 K [16], as opposed to similarly sized micron Al, which ignites near 2100 K [64].

### 4.2. The Effect of Increasing Mg Content on Heat Production

**Figure 9:**
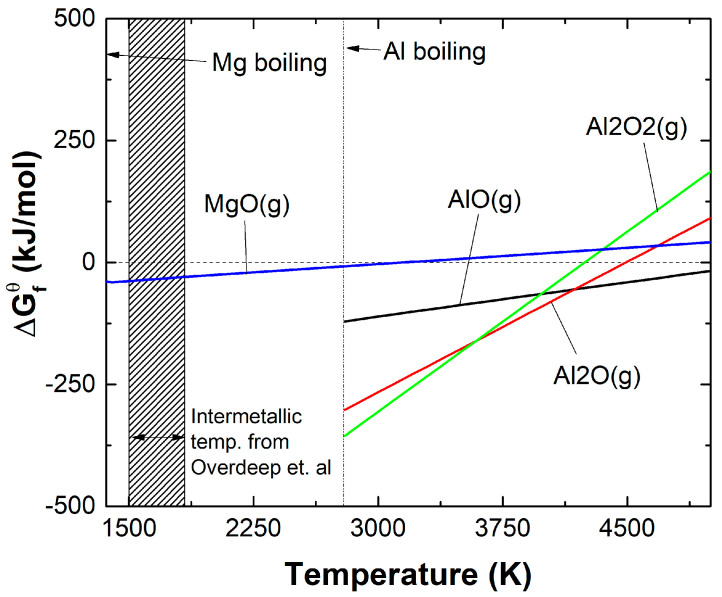
Gibbs free energy of formation curves for possible vapor products plotted from the boiling point of the respective element and beyond. Figure reproduced from the National Institute of Standards and Technology (NIST) JANAF tables [66].

Pure Al and Mg at the micron scale both burn heterogeneously in oxygen through a vapor diffusion flame and demonstrate condensation of nano-scale oxides, which precipitate from the vapor phase [15]. Previous research has shown that these Al/Zr composite particles burn in a dual-phase (vapor oxidation and condensed nitridation/oxidation), which results in particles with a higher extent of combustion than observed here [45]. The overall decrease in heat output from the theoretical value may be a result of this dual-phase combustion not proceeding to completion, as the combustion efficiency of similar Al/Mg/Zr composite particles is 80–90% in a larger chamber under different ignition conditions [21]. Further, given that the addition of Mg does not lower the theoretical heat of combustion significantly, the reduced measured heat of combustion for the Al–38Mg:Zr particles, compared with the other particle compositions, is attributed to other potential sources. Reported experimental evidence suggests that a Mg-dominated burn occurs before Al combustion in mechanically milled Al–Mg composites [48,49]. The same mechanism is likely occurring here owing to the large disparity between the boiling points of Al (2743 K) and Mg (1383 K) [65]. That is, Mg may be preferentially boiling out of the composites during the intermetallic formation reaction, which reaches temperatures ranging from 1500 to 1800 K [46]. Significant Al evaporation and oxidation will only begin as the particles continue to heat to higher temperatures near 2743 K. At that stage, Al vapor will oxidize preferentially over any remaining Mg vapor, as suggested by the thermodynamic data in Figure 9 [66]. Curves above the 0 kJ/mol dotted line in Figure 9 will not form spontaneously. Figure 9 highlights the driving forces for the various gas phase oxidative processes that are occurring, but does not replace a more nuanced understanding of the burn mechanism of these complex composites.

**Figure 10:**
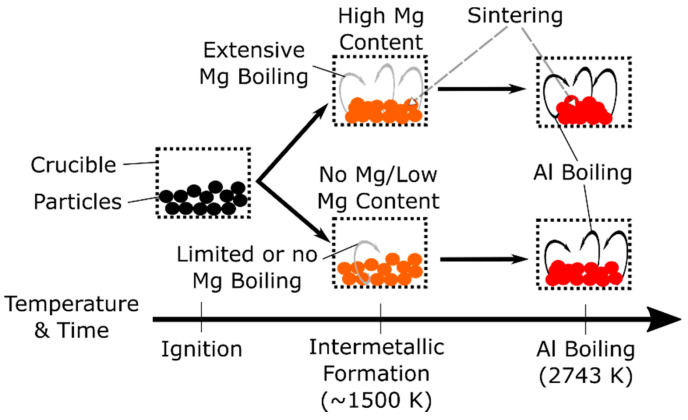
A temperature-time schematic of the transition in flame species, which is present in higher Mg content particles, but not in particles that have low or no Mg content.

Our observation that the residue on the product particles contains both Al and Mg oxides implies that Al and Mg are vaporizing at least somewhat concurrently; that is, the diffusion flame commonly seen for pure Mg and Al may, in this case, be an intermixed flame of both Al and Mg species where the concentration of the metal in the flame may change in time. In other words, there may be a gradual transition from Mg to Al vaporization and oxidation. In support of these observations, complex Al–Mg spinel oxides have been observed in large-scale explosively dispersed combustion experiments with similar Al–Mg–Zr composite powders, implying that Al and Mg may be burning concurrently in the vapor state [21]. The increasing presence of MgO in the diffraction pattern with increasing Mg content (Figure 8) provides some indirect evidence of Mg oxidizing to form MgO prior to Al oxidation. This oxidation of Mg in the vapor state, early in the combustion process, is likely radiatively heating the particles and causing an additional stage of sintering prior to Al boiling. This effect would increase for larger Mg concentrations and may cause the particles to fold and buckle in on themselves (as observed for foils in [46]), further reducing the active surface area of the particles. Such behavior is depicted schematically in Figure 10. This proposed sintering effect would be mitigated with enhanced particle dispersal. Other phenomena such as particle cooling owing to Mg evaporation may also be active, as suggested in previous work [46]. These combined effects could explain the lower combustion efficiencies that are observed for the Al–38Mg:Zr samples.

The predicted sintering effect may also explain why the ball milled particles produce less heat than the PVD particles for the Al–38Mg:Zr samples, as shown in Figure 4. The ball milled particles have a roughly spherical shape and a higher packing fraction that is predicted to approach 60% [67,68], while the half-pipe shaped 40 µm PVD particles were measured to have packing fractions ranging from 37% to 40% (see Appendix A and Table S1 in the Supplementary Materials for details.) The higher packing fraction leads to more interfacial contact area and a lower void content within the initial sample powder bed for the ball milled powders, both of which can enhance sintering. More sintering of the ball milled powders would in turn reduce the extent of combustion, and hence the combustion efficiency of these powders relative to the PVD powders.

Ultimately, more *in situ* studies of the various reactions (including, for example, dynamic pressure measurements during these closed-volume experiments or mechanistic studies outside of the bomb chamber) would help to elucidate the reaction mechanisms. Though more information is needed to confirm these processes, it is clear that particle dispersion and oxygen availability play a large role in the heat produced by these burning composites. In various applications of these particles, there may be advantages to adding Mg, such as a decreased ignition delay, an increase in the burn rate, and benefits of MgO production for bio- and chemical-agent defeat. Future iterations of these experiments will include experimental improvements such as adding dispersants, utilizing higher pressures, and leveraging thermite mixtures.

## 5. Conclusions

Al:Zr, Al–8Mg:Zr, and Al–38Mg:Zr composite particles were fabricated by both PVD and ball milling and reacted at 1 atm of O_2_ in a custom, highly sensitive micro-bomb calorimeter. It was found that Al:Zr and Al–8Mg:Zr particles, whether sputtered deposited or ball milled, outperform the combustion efficiency of thin-film foils of the same composition by 30% or more. However, the measured values were ~28–57% lower than the theoretical maxima, which is attributed to sintering of the particles and incomplete combustion. Sintering is also thought to explain why an effect of particle size on the measured heats of combustion was not observed. A decrease in total energy output was measured for the high Mg-content Al–38Mg:Zr particles relative to the particles of other compositions. This reduction is attributed to increased vaporization of Mg and a reduction in the active surface area owing to increased sintering, folding, and buckling of the particles, as observed for foils in previous work. Finally, physically mixed Al and Mg nano-oxide combustion products were observed, condensed onto the surface of the particles, which implies these materials may provide benefits for bio- and chemical-agent defeat applications.

## Figures and Tables

**Table 1:** The mass of fuel needed to consume 100% of available O_2_ in the calorimeter, assuming 1 atm of O_2_, complete combustion, and ideal gas behavior in a calorimeter with a volume of 15 cm^3^.

Composition	Mass (mg)
Al:Zr	44.76
Al–8Mg:Zr	45.2
Al–38Mg:Zr	46.95

**Table 2:** Measured sizes and heat produced by burning Al:Zr PVD particles of varying size, using surface-area-to-volume ratio (SA/V) calculated from Equation (1).

Particle Size	Avg. Particle Width (µm)	Avg. Particle Length (µm)	Avg. Particle Thickness (µm)	Avg. SA/V	Heat (kJ/g)
“Small”	40 ± 6	145 ± 5	10 ± 6	0.083	10.2 ± 1.3
“Standard”	68 ± 5	141 ± 7	44 ± 5	0.048	11.5 ± 1.6
“Large”	84 ± 6	204 ± 9	52 ± 6	0.038	11.7 ± 1.0

## Supplementary Materials

The following are available online at https://www.mdpi.com/1996-1944/13/12/2745/s1, Figure S1: spherical reaction products collected outside reaction crucible, Figure S2: post-reaction products collected inside reaction Crucible, Figure S3: spectroscopy of Al/Mg/Zr composites mixed with an oxide burning in air, Table S1: packing density for PVD particles of varying thicknesses.

## Appendix A

In order to decouple trends associated with particle size and packing density, the theoretical maximum density (i.e., %TMD or packing fraction) of loosely-poured PVD powders was measured. The packing fraction was expected to scale with the length/thickness ratio of the PVD particles. Optical microscopy (Leica DMi8A, Buffalo Grove, IL, USA) was used to characterize the length and width size distributions of the PVD particles, while packing density was measured by pouring the particles into a 10 mL graduated and measuring the associated mass. To maintain consistency, the cylinder was not agitated, and as-poured measurements were performed three times to obtain average values. The cylinder was of large enough diameter that any interactions between the wall and particles were insignificant.

PVD particle packing densities are listed in Supplementary Materials, Table S1 and the %TMD for each PVD particle size is calculated as the packing density of the loose compact relative to the density of a fully dense alloy of the same composition. The loose particle compacts have a %TMD between 37% and 40%, except for the thinnest Al:Zr particles, for which the %TMD is only 29.7%. The smaller %TMD is attributed to a larger length to thickness ratio of 14.5:1 for the 20 µm thick particles. The other samples (intermediate and large sizes) have length to thickness ratios of 5.8:1 and 6.4:1, respectively
