# Supplementary material for: Measuring Heat Production from Burning Al/Zr and Al/Mg/Zr Composite Particles in a Custom Micro-Bomb Calorimeter
Source: Materials (Basel). 2020 Jun 17;13(12):2745. doi: 10.3390/ma13122745 (PMC7345939; doi:10.3390/ma13122745)
Supplement: Supplementary file 1 [file materials-13-02745-s001.pdf]

materials

Supplementary Materials

Measuring Heat Production from Burning Al/Zr and Al/Mg/Zr Composite Particles in a Custom Micro-Bomb Calorimeter

Elliot R. Wainwright *,†, Madeline A. Mueller †, Kyle R. Overdeep †,
Shashank Vummidi Lakshman and Timothy P. Weihs *

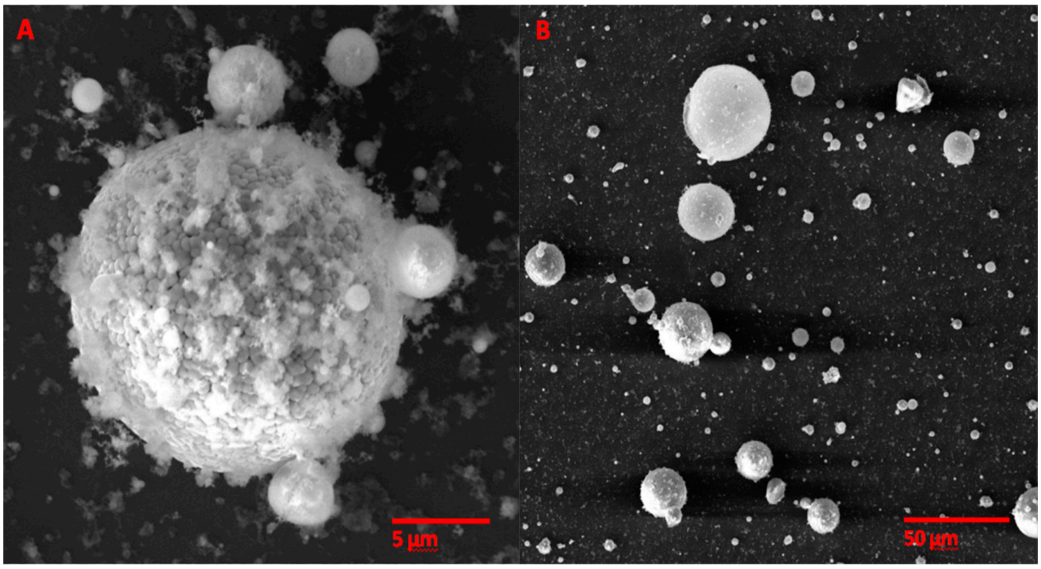

Figure S1: (A,B) Secondary electron micrographs of spherical reaction products for 40 µm thick Al–8Mg:Zr PVD particles, collected post-reaction from the bottom of the calorimeter chamber. The spherical nature of the particles suggest a molten state during reaction and the presence of fine soot suggests the evaporation and oxidation of Al and Mg.

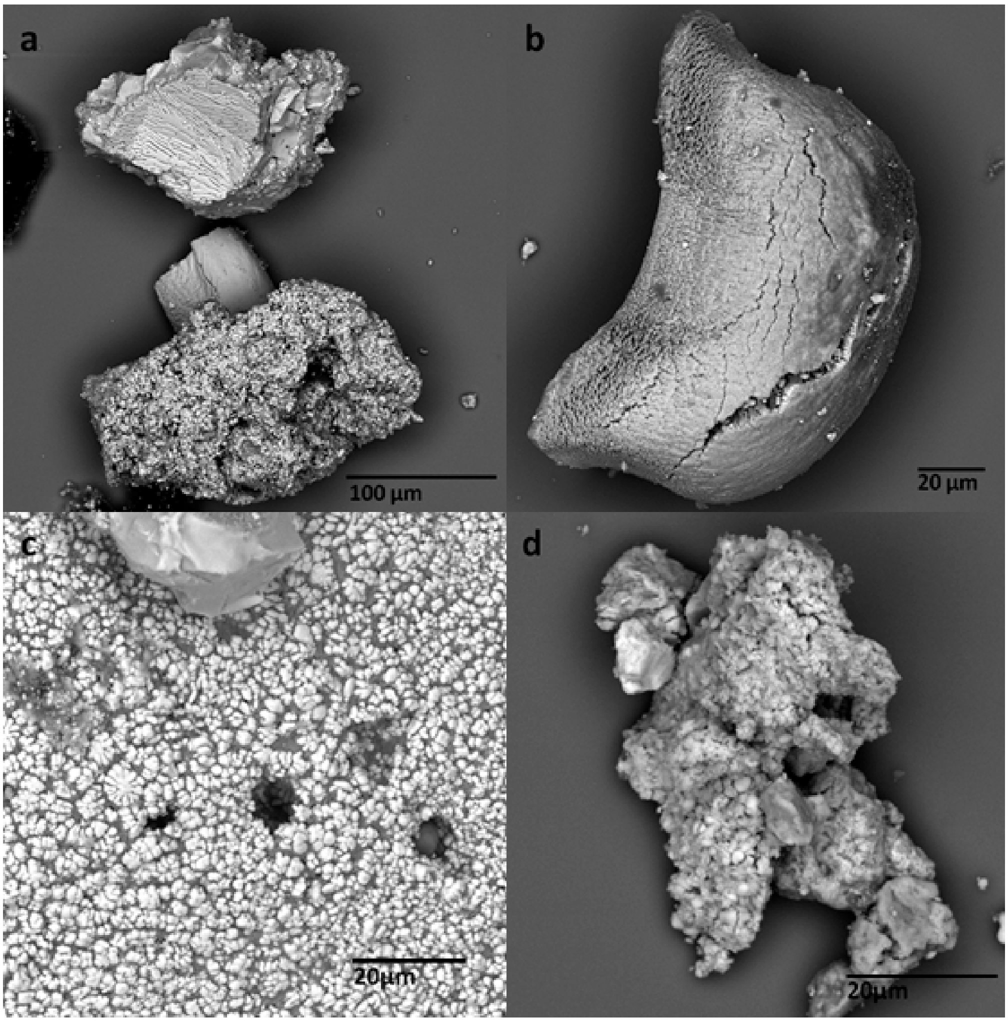

Figure S2: (a,b) Backscatter electron micrographs of post-reaction products collected inside the crucible. **(a)** PVD Al:Zr 40 µm particles have agglomerated into clumps or exhibit rough, unreacted edges. **(b)** A single, partially oxidized PVD Al:Zr 40 µm particle that quenched after reacting and forming an intermetallic phase but before combusting at high temperatures. **(c)** Image of the surface of a reacted Al–8Mg:Zr 40 µm PVD particle showing phase separation of the Al and Zr oxidizes. Lighter regions are Zr-rich, while darker regions are Al-rich. **(d)** Post- reaction ball milled Al–8Mg:Zr particles sintered together.

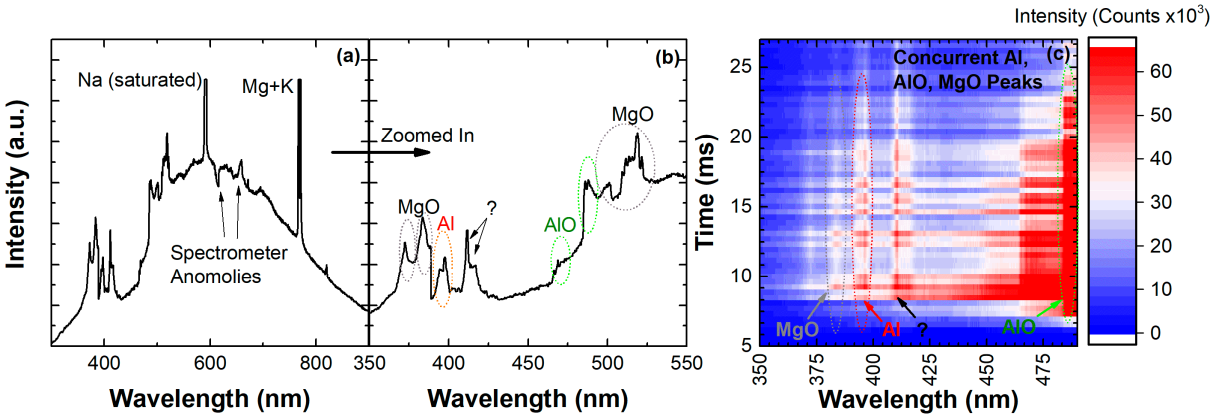

Figure S3: (**a**) A snapshot from a time-resolved optical spectroscopy trace, collected from a one inch region above a reaction crucible mixed with 4(Al–8Mg):Zr (i.e. 73.6 at% Al, 6.4 at% Mg, 20 at% Zr) and an HIO_3 oxidizer for dispersion, burning in air. The spectrometer is an VIS/NIR Avantes AvaSpec-3648. (**b**) a magnified image of the features between 350 and 550 nm. The figure demonstrates multiple AlO and MgO peaks implying Al and Mg burning in the vapor state. (**c**) A top-down intensity map of the spectra in time, unsubtracted from its natural grey-body background. Though this is an indirect comparison to the experimental system utilized in this work, these traces show that during the combustion of these particles Al, AlO, Mg, and MgO peaks are present.

Table S1: Packing density for PVD particles of varying thicknesses and associated sizes.

Composition and Size Designation	Target Particle Thickness (µm)	Actual Avg. Particle Thickness (µm)	Packing Density (g/cm³)	Theoretical Density (g/cm³)	%TMD	Length: Thickness Ratio
Al:Zr "Small"	20	10 ± 6	1.46 ± 0.02	4.928	29.7	14.5:1
Al:Zr "Standard"	40	44 ± 5	1.84 ± 0.05	4.928	37.3	5.8:1
Al:Zr "Large"	60	52 ± 6	1.97 ± 0.02	4.928	40.0	6.4:1
Al–8Mg:Zr	40	44 ± 5	1.91 ± 0.04	4.855	39.4	5.8:1
Al–38Mg:Zr	40	44 ± 5	1.73 ± 0.02	4.596	37.7	6.4:1
